# Comparison of two polymerase chain reaction assays, DaAn Gene (DaAn Gene 2019-ncov) and Cepheid (Cepheid Xpert Xpress SARS-CoV-2) for Delta and Omicron variants of SARS-CoV-2 detection in Yaoundé, Cameroon

**DOI:** 10.4102/jphia.v16i1.1282

**Published:** 2025-07-31

**Authors:** Martin Maidadi-Foudi, Marie Atsama-Amougou, Celestin Godwe, Emilande Guichet, Nadine L. Boutgam, Guy Roland Awoundza Metomo, Oumarou Goni Hamadama, Gilles-Fils Woguia, Yannick F. Ngoume, Dowbiss Meta Djomsi, Roméo Brice D. Djounda, Modeste Ngamaleu, Hillary Tene, Livo Esemu, Joseph Fokam, Marie-Claire Okomo, Boyomo Onana, Wilfred Mbacham, Marcel Tongo, Eric Delaporte, Martine Peeters, Ahidjo Ayouba, Charles Kouanfack

**Affiliations:** 1 Centre for Research on Emerging and Re-emerging Diseases (CREMER), Institute of Medical Research and Medicinal Plants Studies (IMPM), Yaoundé, Cameroon; 2 Department of Microbiology, Faculty of Science, University of Yaoundé I, Yaoundé, Cameroon; 3 Department of Biochemistry, Faculty of Science, University of Douala, Douala, Cameroon; 4 Translational Research on HIV and Endemic and Emerging Infectious Diseases (TransVIHMI), University of Montpellier/Institute of Research for Development (IRD), Montpellier, France; 5 Virology Laboratory, Chantal Biya International Reference Centre (CIRCB) for Research on HIV/AIDS Prevention and Management, Yaoundé, Cameroon; 6 Faculty of Medicine and Biomedical Sciences, University of Yaoundé I, Yaoundé, Cameroon; 7 National AIDS Control Committee, Ministry of Public Health, Yaoundé, Cameroon; 8 National Public Health Laboratory (NPHL), Ministry of Public Health, Yaoundé, Cameroon; 9 Biotechnology Centre of Nkolbisson (CBT), University of Yaoundé I, Yaoundé, Cameroon

**Keywords:** mutations, severe acute respiratory syndrome coronavirus 2, reverse transcription-polymerase chain reaction, delta variant, omicron variant, Cameroon

## Abstract

**Background:**

New severe acute respiratory syndrome coronavirus 2 (SARS-CoV-2) variants may affect diagnostic test accuracy.

**Aim:**

To evaluate the performance of two reverse transcription-polymerase chain reaction (RT-PCR) assays, DaAn Gene and Cepheid, for detecting Delta and Omicron variants.

**Setting:**

Nasopharyngeal samples were collected in Yaoundé, Cameroon, between October 2021 and December 2022.

**Methods:**

Nasopharyngeal samples were tested with both assays. Samples with a cycle threshold (CT) ≤ 28 were sequenced. Cohen’s kappa coefficient assessed assay agreement.

**Results:**

We compared 294 samples. At CT ≤ 40, DaAn Gene showed a 59.2% (*n* = 174/294) positivity rate versus 57.8% (*n* = 170/294) for Cepheid (χ^2^ = 0.695, *p* = 0.4044). Agreement was 95.91% (*n* = 282/294) with κ = 0.95. DaAn Gene showed a 58.2% (*n* = 171/294) positivity rate versus 54.4% (*n* = 160/294) for Cepheid at CT ≤ 37 (*p* = 0.5058), with 95.6% agreement (κ = 0.95). At CT ≤ 33, DaAn Gene was 56.5% (*n* = 166/294) positive versus 54.1% (*n* = 159/294) for Cepheid (*p* = 0.6187), with 96.93% agreement (κ = 0.938). For 167 samples with CT ≤ 28, agreement was 97.0% (κ = 0.97) across variants. These RT-PCR assays effectively detected Delta and Omicron variants.

**Conclusion:**

The emergence of Delta and Omicron variants did not significantly impact the diagnostic performance of these routine RT-PCR assays.

**Contribution:**

This study confirms their continued effectiveness in detecting these variants in this setting.

## Introduction

A widespread viral pneumonia epidemic emerged at the end of 2019 in Wuhan, China, caused by the severe acute respiratory syndrome coronavirus 2 (SARS-CoV-2).^1^ Extremely transmissible, SARS-CoV-2 had spread rapidly around the world, posing an extraordinary threat to global public health. Given the contagious nature of SARS-CoV-2, it was imperative to have rapid, sensitive and accurate diagnostic methods to easily detect the virus and reduce its spread.^2^ Screening tests for SARS-CoV-2 infection can be divided into three groups: tests to detect the presence of viral ribonucleic acid (RNA) by reverse transcription-polymerase chain reaction (RT-PCR) or other amplification techniques, tests to detect viral antigens and analyses of antibodies to SARS-CoV-2 antigens.^3^ Real-time RT-PCR is the method of choice for diagnosing current or active infection with SARS-CoV-2.^4^ In the year 2020, various commercially available kits were developed for the detection of SARS-CoV-2 using RT-PCR. The spike (S), RNA-dependent RNA polymerase (RdRp), nucleocapsid (N) and envelope (E) genes, as well as the functionally important open reading frames 1a and 1b (ORF1ab), are the polymerase chain reaction (PCR) targets of the available kits. Most of these kits are multiplexed to detect at least two of the above-mentioned genes.^5^ In Cameroon, several RT-PCR test kits were used to diagnose SARS-CoV-2 infection, including the Abbott RealTime SARS-CoV-2 targeting RdRp and N genes, the DaAn Gene kit targeting the N and ORF1ab genes and the Cepheid assay detecting the N2 region of the SARS-CoV-2-specific nucleocapsid (N) gene and the pan-sarbecovirus E gene.

Although the SARS-CoV-2 virus has exhibited considerable genetic diversity globally throughout the COVID-19 pandemic, the fundamental characteristics of the strain have remained largely consistent to be more understandable.^6^ Current figures seem to show that the SARS-CoV-2 genome acquires variability at a rate close to 9.8 × 10^-4^ changes per site per year.^7^ The high replication rate, spread and frequency of the virus are correlated with new viral variants, and these features depend on mutations in the viral genome. Mutagenesis, especially in the S1 subunit of the spike protein, contributes to the virulence, infectious power and spread of the virus.^8^ While changes in the spike gene are the main feature of the variants of concern (VOC) and variants of interest (VOI) lineages, the detection of other mutations in other parts of the genome requires a more complete examination to measure the possible effects on the analytical capabilities of diagnostic tests. Diagnostic tests were designed to identify segments of the virus that are, in principle, well conserved. It is therefore crucial to continue to evaluate these assumptions by analysing sequences and samples to ensure that they remain in phase with viral evolution during the SARS-CoV-2 pandemic waves.^9^ Since viruses continue to evolve, diagnostic tests need to remain effective and detect the increasing number of circulating variants.^10,11,12^ While the design of tests takes viral diversity into account, their performance will need to be constantly assessed in the light of the variants detected and through molecular surveillance.^9,13,14,15^ In this study, we evaluated the performance of the Cepheid RT-PCR kit and DaAn Gene RT-PCR kit used in routine diagnosis in Cameroon to detect the Delta and Omicron variants of SARS-CoV-2. The DaAn Gene RT-PCR kit was the most used in all laboratories in Cameroon, firstly because of its availability and accessibility and secondly because of its ease of use. The evaluation of the DaAn Gene RT-PCR kit is crucial because of the genetic variability introduced by the dominant Delta and Omicron SARS-CoV-2 variants.^16,17^ These mutations, can potentially compromise the sensitivity of RT-PCR tests, leading to false negatives.^18,19,20^ Understanding the impact of these variants is also essential because their differing transmissibility and severity influence the pandemic’s dynamics, requiring effective diagnostic tools. A reliable test such as DaAn Gene is vital for accurate and rapid identification of positive cases, aiding public health decisions and pandemic management.^21,22,23^ Therefore, assessing the kit’s performance against Delta and Omicron ensures continued accurate and rapid diagnosis amidst the evolving genetic landscape of SARS-CoV-2.

## Research methods and design

### Study design

This was a comparative cross-sectional study of two RT-PCR protocols, DaAn Gene (DaAn Gene Co. Ltd. of Sun Yat-sen University, China)^24^ and Cepheid (Cepheid, France)^25^ routinely used for the diagnosis of SARS-CoV-2 in Cameroon. Nasopharyngeal samples were tested in pairs using both molecular diagnostic techniques for the detection of SARS-CoV-2. Samples that tested positive for cycle threshold (CT) value ≤ 28 were subsequently sequenced by Illumina next-generation sequencing (NGS) (iSeq 100) Instrument using COVIDSeq kit (Illumina Corp., San Diego, United States [US]).^26^

### Study sites and population

This study was conducted at the Centre for Research on Emerging and Re-emerging Diseases (CREMER) in Yaoundé. It focused on nasopharyngeal samples collected between 08 October 2021 and 16 December 2022 during successive waves of Delta and Omicron variants. Samples were collected at the CREMER sampling site and also from sampling sites set up by the ‘Centre Régional de la Santé Publique (DRSPC)’. These included the district hospitals of Nkolndongo, Nkolbisson, the ‘Palais polyvalent et des Sports de Yaoundé (PAPOSY)’ and the DRSPC.

### Sample collection and processing

All clinical samples were collected from individuals tested for COVID-19 diagnosis in Yaoundé. Briefly, nasopharyngeal swabs were collected, by trained personnel, in a 1 mL tube containing viral transport medium (Suzhou Cellpro Biotechnology Co., Ltd) according to the manufacturer’s instructions and under universal biosafety measures. The samples were stored between 4 °C and 8 °C for 2 h – 5 h and referred to CREMER for laboratory processing.

### Laboratory procedures at Centre for Research on Emerging and Re-emerging Diseases

#### Nucleic acid extraction, amplification and detection with DaAn Gene protocol

For DaAn Gene RT-PCR protocol, nucleic acid was manually extracted from 200 µL nasopharyngeal swab using the ‘DNA/RNA purification kit’ as per manufacturer’s instructions.^24^ The amplification and detection were performed using the detection kit for 2019 novel coronavirus (2019-nCoV) RNA (PCR-Fluorescence Probing) (DaAn Gene Co. Ltd., China)^24^ on the QuantStudio 5 Thermocycler (Thermofisher Scientific). The protocol used probes targeting the open reading frame (ORF1ab) gene and the nucleocapsid (N) protein gene, with a lower limit of detection of 500 copies/mL and an amplification reaction of 45 cycles as per the manufacturer’s instructions; each sample with a CT ≤ 40 was considered positive, while a CT > 40 or ‘undetermined’ (no amplification after 45 cycles) was considered negative.

#### Nucleic acid extraction, amplification and detection with Cepheid protocol (point of care)

The Cepheid system automates and integrates sample preparation, nucleic acid extraction, amplification and detection of target sequences in simple or complex samples by real-time PCR. We used 300 µL of nasopharyngeal samples directly loaded into the Cepheid cartridge and passed directly to the GenXpert (automated system).^25^ Two genes were targeted in this protocol (N2 and E genes). The Cepheid assay gave positive results when a signal of N2 gene or signals of both N2 and E genes had a CT within the valid range (CT < 45) and the endpoint above the minimum setting. A presumptive positive result was given when only a signal of E gene has been detected. Negative results were given when none of N2 or E genes were detected.

#### Whole-genome sequencing, bioinformatics and phylogenetic analysis

Full-genome sequencing on the Illumina Platform (iSeq100) was performed on all samples confirmed positive by RT-quantitative polymerase chain reaction (qPCR) with a CT value ≤ 28. The Illumina COVIDSeq assay kit was used following the manufacturer’s protocol^12^ for complementary deoxyribonucleic acid (cDNA) synthesis, amplification as well as library preparation. The reads assembly and the variants and/or lineages assignment were carried out by the GeVarLi Pipeline (GeVarLi: GEnome assembly, VARiant calling and LIneage assignment).^27^ Mutation calling was carried out using the Coronavirus Resistance Database of Stanford University system.^28^

### Statistical analyses

Statistical analyses were performed using IBM^®^ SPSS^®^ statistics version 25 software. The concordance of the diagnosis tests was evaluated according to Cohen’s kappa (*k*) value, and the logical interpretation of *k* according to the criteria proposed by McHugh in 2012: *k* = 0.01–0.20 (none agreement), *k* = 0.21–0.39 (minimal agreement), *k* = 0.40–0.59 (weak agreement), *k* = 0.60–0.79 (moderate agreement), *k* = 0.80–0.90 (strong agreement) and *k* above 0.90 (almost perfect agreement).^29^ The diagnostic concordance was then compared according to different CT values (according to the manufacturer’s instructions at CT ≤ 40, Cameroon National Algorithm at CT ≤ 37 and the threshold for SARS-CoV-2 transmissibility at CT ≤ 33). We used a chi square test to compare the performances of the two assays. Finally, we determined diagnostic concordance across variants (Delta and Omicron). All *p*-values < 0.05 were considered statistically significant, with a 95% confidence interval (CI).

### Ethical considerations

This study benefited from an authorisation for the use of Centre for Research on Emerging and Re-emerging Diseases (CREMER) data and samples, an authorisation from the Ministry of Public Health within the framework of an agreement between this Ministry and CREMER for the Genomic Surveillance of SARS-CoV-2 (AFROSCREEN Project) and an ethical clearance N°2020/05/1218CE/CNERSH/SP from the ‘Comité National d’Ethique de la Recherche pour la Santé Humaine (CNERSH)’.

## Results

### Description of the selected samples

**FIGURE 1:**
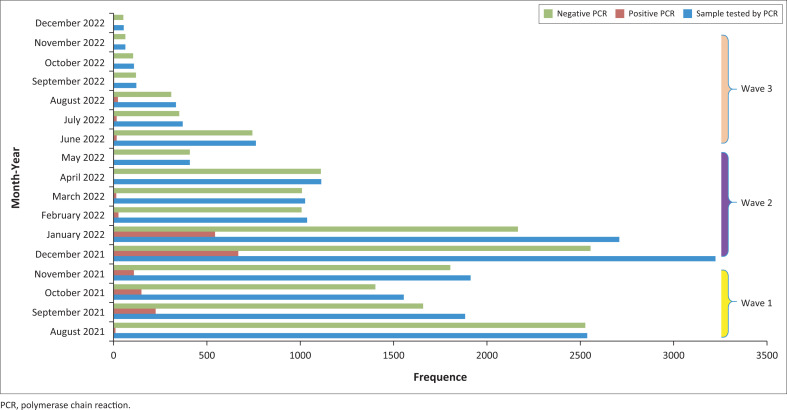
Severe acute respiratory syndrome coronavirus-2 reverse transcription-polymerase chain reaction tests performed per month during the study period.

During our study period between August 2021 and December 2022, a total of 19 237 nasopharyngeal samples were tested by RT-PCR. The tests performed per month ranged from 55 RT-PCR tests in December 2022 to 3225 RT-PCR tests in December 2021 (Figure 1).

**FIGURE 2:**
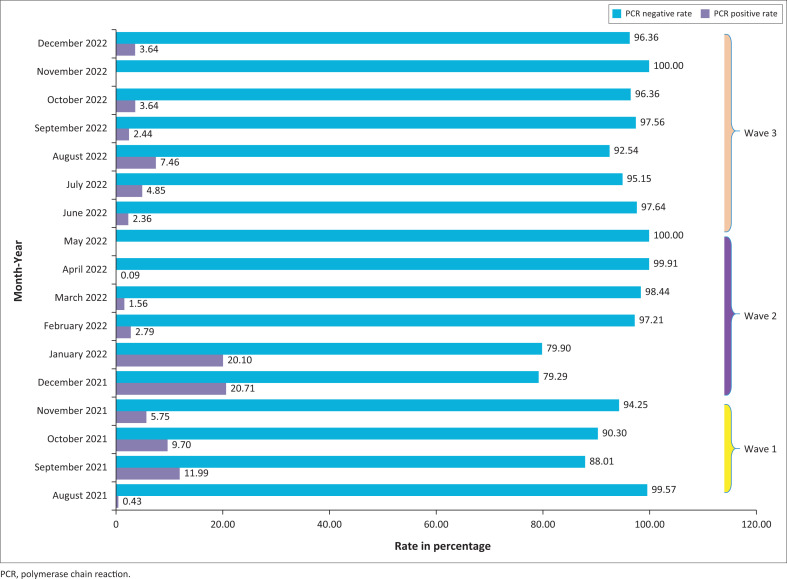
Positivity rate of severe acute respiratory syndrome coronavirus-2 polymerase chain reaction within the study period.

During that period, PCR test positivity rates varied from month to month in the city of Yaoundé. They ranged from 0.0% in May 2022 and November 2022 to 21.0% in December 2021. During this study, we noted three variations that we described as three different waves: firstly, Wave 1 between August 2021 and November 2021 with a peak in September 2021 at 12%; secondly, Wave 2 between December 2021 and May 2022 with a peak in December 2021 at 21.0%; thirdly, Wave 3 between June 2022 and November 2022 with a peak in August 2022 at 7.46% (Figure 2).

In this study, we initially enrolled 315 nasopharyngeal samples from suspected SARS-CoV-2 positive individuals (Table 1). By strictly applying the criteria for interpreting the results of the two protocols (CT ≤ 40 for DaAn Gene and 45 for Cepheid), we obtained the following results: four samples presumed positive with Cepheid compared to 17 with DaAn Gene test and 294 samples had a clearly defined status (positive or negative) by both protocols. There was no concordance between the four presumptive positives obtained by Cepheid vs DaAn Gene protocols. Among the four samples, three were positive and one was negative with the DaAn Gene. Similarly, the results of the 17 presumed positive samples obtained by the DaAn Gene manual method did not agree with the results obtained by the point-of-care method, Cepheid protocols. Of the 17 samples, 3 were positive and 14 were negative with DaAn Gene method. In view of the discrepancy in these results, we excluded the 21 samples from further analysis. The comparison of these two protocols was made only on the 294 samples for which the status of their results was clearly qualified as positive or negative. Out of the 294 participants enrolled in this study, 48.6% (*n* = 143/294) were males, 48.0% (*n* = 141/294) were females and 3.4% (*n* = 10/294) lacked gender data. Participants in this study ranged in age from 5 years to 79 years, with an average age of 40.3 years. Participants in the 0–20 years age group were the most represented (46.3%), followed by the 21–40 years age group (35.4%).

**TABLE 1:** Reverse transcription-polymerase chain reaction results by DaAn Gene and Cepheid.

RT-qPCR assays	DaAn Gene			
	Negative	Positive	Presumed positive	Total
**Cepheid**				
Negative	116	8	14	138
Presumed positive	1	3	0	4
Positive	4	166	3	173

**Total**	**121**	**177**	**17**	**315**

### Performance of molecular diagnosis tests, DaAn Gene and Cepheid for the detection of SARS-CoV-2 in Yaoundé

Of the 294 samples selected for the concordance analyses between the two protocols with clearly defined status (positive or negative) and whose results were returned to patients, we studied the concordance for CT values harmonised at 40, 37 and 33. At the CT ≤ 40, the DaAn Gene SARS-CoV-2 real-time test showed a positivity rate of 59.2% (*n* = 174/294) compared to 55.4% (*n* = 163/294) for the Cepheid test (χ^2^ = 0.695, *p* = 0.4044). The overall percentage agreement between these two tests was 95.58% (*n* = 281/294), with a Cohen’s kappa value of *k* = 0.910 (95% CI), indicating almost perfect agreement between the two diagnostic protocols. If we reduce this positivity threshold to a CT ≤ 37 for the two protocols as defined in the national algorithm for the diagnosis of SARS-CoV-2 in Cameroon, we obtain a positivity rate of 58.2% (*n* = 171/294) for the DaAn Gene protocol compared to 55.1% (*n* = 162/294) for the Cepheid protocol (χ^2^ = 0.443, *p* = 0.5058). The overall percentage of agreement between the two protocols remained similar, at 96.93% (*n* = 285/294) with a Cohen’s kappa value of *k* = 0.938 indicating almost perfect concordance between the two protocols. If we reduce this positivity threshold to a CT ≤ 33 corresponding to the limit of the transmissible viral load for the two protocols, we obtain a positivity rate of 56.5% (*n* = 166/294) for the DaAn Gene protocol compared to 54.1% (*n* = 159/294) for the Cepheid protocol (χ^2^ = 0.248; *p* = 0.6187). At a CT threshold value of 37, the overall percentage agreement between the two protocols remained similar at 96.93% (*n* = 285/294), with a Cohen’s kappa value of *k* = 0.938 indicating almost perfect agreement between them (Table 2).

**TABLE 2:** Concordance between the DaAn Gene and Cepheid protocols for the molecular diagnosis of SARS-CoV-2.

CT threshold value	Cepheid			SARS-CoV-2 molecular diagnostic protocols						Cohen’s kappa value (*k*)
				DaAn Gene			Overall % of concordance			
	%	*n*	*N*	%	*n*	*N*	%	*n*	*N*	
CT positivity rate < 40	55.4	163	294	59.2	174	294	95.58	281	294	0.910
CT positivity rate < 37	55.1	162	294	58.2	171	294	96.93	285	294	0.938
CT positivity rate < 33	54.1	159	294	56.5	166	294	96.93	285	294	0.938

SARS-CoV-2, severe acute respiratory syndrome coronavirus-2; CT, cycle threshold.

### Concordance between the DaAn Gene and Cepheid protocols according to the SARS-CoV-2 variant detected by complete genome sequencing on the Illumina platform

From the 174 samples declared positive by the DaAn Gene protocol, we obtained 164 sequences of SARS-CoV-2 full genome, including 26.83% (*n* = 44/164) Delta variants and 73.17% (*n* = 120/164) of Omicron variants. The positive concordance of the DaAn Gene and Cepheid protocols for the detection of Delta and Omicron variants was 88.6% (*n* = 39/44) and 97.5% (*n* = 117/120), respectively. All 44 Delta variant samples were 100% detected by the DaAn Gene kit, while 5 samples were not detected by the Cepheid PCR kit. Similarly, all 120 Omicron variant samples were detected by the DaAn Gene kit, while 3 Omicron samples were not detected by the Cepheid PCR kit (Table 3).

**TABLE 3:** Distribution of results obtained by the DaAn Gene and Cepheid polymerase chain reaction techniques depending on the variants detected.

Variant	Cepheid positive	Cepheid negative	Total DaAn Gene
**Delta**			
DaAn Gene positive	39	5	44
DaAn Gene negative	0	0	0
Total Cepheid	39	5	44
**Omicron**			
DaAn Gene positive	117	3	120

## Discussion

Detecting emerging SARS-CoV-2 VOCs early in healthy carriers and patients before they spread the virus to others can have a major impact on triggering sudden epidemics.^30^ Reverse transcription-polymerase chain reaction tests are the most widely used for detecting SARS-CoV-2 and COVID-19 in clinical samples.^31,32,33^ Polymerase chain reaction techniques can detect specific portions of genomic RNA and identify SARS-CoV-2 with high sensitivity and specificity.^34^ Generally, the assay is developed from the sequences of the whole genome at the time of conception, which means that all the target species can be included.^34^ On the contrary, the rapid accumulation of mutations in viral genomes as the population increases can lead to signature degradation and failures in analysis, resulting in grey areas during a pandemic.^35^ The real-time RT-PCR tests used in the field (point of care), in addition to the conventional platform, can contribute to the early, sensitive and specific detection of SARS-CoV-2.^22^

In the present study, we assessed the concordance of a conventional assay (DaAn Gene) and an automated ‘Point-of-Care’ (POC) real-time RT-PCR assay (Cepheid) for the detection of the VOC Delta and Omicron of SARS-CoV-2 in nasopharyngeal samples. Cepheid, which uses probes targeting both the E and N2 genes of SARS-CoV-2, is one of the real-time RT-PCR techniques that can detect SARS-CoV-2 in 50 min, with detection limits of 100 copies/mL.^25^ The conventional real-time RT-PCR test (DaAn Gene), which serves as the basic technique, uses probes targeting the open reading frame gene (ORF1ab) and the nucleocapsid protein gene (N), with a lower limit of detection of 500 copies/ml.^36,37^ Polymerase chain reaction assays are susceptible to false negatives resulting from mutations that weaken primer coupling.^37,38,39^ In our study comparing these two assays during successive waves of Delta and Omicron variants, we obtained almost perfect agreement between the two protocols for the diagnosis of SARS-CoV-2 by considering the manufacturer’s threshold (CT < 40), as well as locally adopted (CT < 37) and transmissibility threshold (CT < 33). Discordance was observed at high CT values and therefore low viral load. Fainguem et al., in their study in 2020, during the first wave of the SARS-CoV-2 epidemic in Cameroon, observed a high degree of concordance in the detection of SARS-CoV-2 between automated (Abbott m2000) and manual (DaAn Gene) RT-PCR systems at CT values of ≤ 37 and ≤ 35.^40^ However, they pointed out that the difference appeared when the viral load was low, highlighting the effectiveness of these tools in limiting the spread of SARS-CoV-2 within the community.^40^ Discordances were noted at high CT values, and this omitted detection may have not been a factor in the spread of the virus, as infected patients who had respiratory samples with CT values above 33–34 were not contagious. However, caution would be needed if the patient was in an early phase after exposure to the virus, as their viral load may subsequently increase.^41^ The threshold CT value of ≤ 37 recommended in the national SARS-CoV-2 screening algorithm seemed to be the golden mean that discriminates between patients with a transmissible viral load and those who were no longer contagious, in order to avoid unnecessary confinement. However, it would be important to reduce this value to a threshold CT value of 33, which corresponds to the contagiousness threshold of the virus.^41^ In addition, the two protocols detected both Delta and Omicron variants. The work published in the journal ‘Nature reports’ on the genomic surveillance of SARS-CoV-2 in Cameroon showed that the variants circulating during the study period mainly included ancestral lineages (74%), followed by the Delta (15%), Omicron (6%), Alpha (3%) and Beta (2%) variants between 2020 and 2022.^42^

In this work, the DaAn Gene RT-PCR test was used for initial diagnosis, and samples with high viral loads were selected for sequencing.^42^ This RT-PCR kit typically targets conserved genes such as the N gene and ORF1ab, which makes it potentially capable of detecting ancestral lineages as well as VOC.^37,39^ The DaAn Gene test has numerous advantages for POC use in Cameroon. Firstly, its high sensitivity with a low detection limit (500 copies/mL) is crucial for identifying infections with low viral loads, particularly in early or asymptomatic cases. Secondly, this test has very good diagnostic reliability because by targeting conserved genes, it minimises the risk of false-negative results because of variant mutations. Furthermore, its applicability in resource-limited settings makes it a test that can be used in laboratories equipped with standard PCR machines, which is compatible with the laboratory network in Cameroon. Thirdly, its speed in delivering results, which can be obtained quickly within 1 h, facilitates the immediate management of positive cases.^43,44^ However, this test has some drawbacks that may limit its use for POC in Cameroon. These factors primarily consist of limited infrastructure, especially in certain regions of Cameroon that lack access to PCR laboratories, as shown by the uneven coverage of genomic surveillance (only 30% of regions covered). To this, we can add the high cost of RT-PCR tests, which are generally more expensive than rapid antigen tests, potentially limiting their accessibility in a context of limited financial resources. Finally, the absolute necessity of local validation complicates its use for POC. Indeed, although the test performs well globally, it requires specific evaluation to confirm its ability to effectively detect the Delta and Omicron variants in the local context.^44,45,46^

To conclude this section, we can say that the DaAn Gene test presents significant advantages for use in detecting SARS-CoV-2 variants in Cameroon. However, its effectiveness would depend on local validation against the specific mutations of the VOC. Improved infrastructure and wider access to PCR technologies would be necessary to maximise its utility at the point of service. While the two SARS-CoV-2 detection kits compared here have been shown to produce similar results, it was considered important to take the epidemiological context into account when deciding on the diagnostic protocol.

### Limitation

The impossibility of acquiring a suitable third reference test, which could be the initial RT-PCR test published by Corman et al. in 2020 (in the Eurosurveillance journal), which targets the RNA-dependent RNA polymerase (RdRp) gene. This non-commercial test is known for its high sensitivity, and its CT values were calibrated to assess patient infectivity (e.g. CT < 32). Comparing the CT values of the two commercial assays (DaAn Gene and Cepheid) with this historical PCR assay would provide valuable insights into their relative performance.

## Conclusion

This study demonstrated that the use of a rapid and sensitive real-time RT-PCR platform can help increase screening capacity. The possibility of using POC real-time RT-PCR platforms in the context of individual screening strategies was highlighted, as they can generate data comparable to conventional tests in a shorter timeframe. Our data provide evidence for the use of different real-time RT-PCR platforms for routine detection of SARS-CoV-2 variants and for the feasibility of using POC real-time RT-PCR assays in laboratories at the local level for efficient and rapid diagnosis of COVID-19. The SARS-CoV-2 diagnostic protocols (Cepheid and DaAn Gene) used routinely in Cameroon have shown perfect concordance, both during the wave of Delta and Omicron variants. While technically the two methods are equivalent, considerations such as the quantity of samples and the deadline for results must be taken into account when choosing the appropriate test. The results obtained in our study suggest that they can be applied to other pathogens.

## References

[1] Zhu N, Zhang D, Wang W, et al. A novel coronavirus from patients with pneumonia in China, 2019. N Engl J Med. 2020;382(8):727–733. 10.1056/NEJMoa2001017 31978945 PMC7092803

[2] Hu B, Guo H, Zhou P, Shi ZL. Characteristics of SARS-CoV-2 and COVID-19. Nat Rev Microbiol. 2021;19(3):141–154. 10.1038/s41579-020-00459-7 33024307 PMC7537588

[3] Arena F, Pollini S, Rossolini GM, Margaglione M. Summary of the available molecular methods for detection of SARS-CoV-2 during the ongoing pandemic. Int J Mol Sci. 2021;22(3):1298. 10.3390/ijms22031298 33525651 PMC7865767

[4] Wu SY, Yau HS, Yu MY, et al. The diagnostic methods in the COVID-19 pandemic, today and in the future. Expert Rev Mol Diagn. 2020;20(9):985–993. 10.1080/14737159.2020.1816171 32845192

[5] Iglói Z, Leven M, Abdel-Karem Abou-Nouar Z, et al. Comparison of commercial realtime reverse transcription PCR assays for the detection of SARS-CoV-2. J Clin Virol. 2020;129:104510. 10.1016/j.jcv.2020.104510 32570045 PMC7833356

[6] Van Dorp L, Acman M, Richard D, et al. Emergence of genomic diversity and recurrent mutations in SARS-CoV-2. Infect Genet Evol. 2020;83:104351. 10.1016/j.meegid.2020.104351 32387564 PMC7199730

[7] Duchene S, Featherstone L, Haritopoulou-Sinanidou M, Rambaut A, Lemey P, Baele G. Temporal signal and the phylodynamic threshold of SARS-CoV-2. Virus Evol. 2020;6(2):veaa061. 10.1093/ve/veaa061 33235813 PMC7454936

[8] Flores-Vega VR, Monroy-Molina JV, Jiménez-Hernández LE, Torres AG, Santos-Preciado JI, Rosales-Reyes R. SARS-CoV-2: Evolution and emergence of new viral variants. Viruses. 2022;14(4):653. 10.3390/v14040653 35458383 PMC9025907

[9] Rodgers MA, Olivo A, Harris BJ, et al. Detection of SARS-CoV-2 variants by Abbott molecular, antigen, and serological tests. J Clin Virol. 2022;147:105080. 10.1016/j.jcv.2022.105080 35086043 PMC8770247

[10] Public Health Ontario. Coronavirus disease 2019 (COVID-19) – Variant of concern screening and whole genome sequencing surveillance [homepage on the Internet]. [cited 2025 Apr 14]. Available from: https://www.publichealthontario.ca/en/Laboratory-Services/Test-Information-Index/COVID-19-VoC

[11] Lakshmanan K, Liu BM. Impact of point-of-care testing on diagnosis, treatment, and surveillance of vaccine-preventable viral infections. Diagnostics. 2025;15(2):123. 10.3390/diagnostics15020123 39857007 PMC11763637

[12] Cassedy A, Parle-McDermott A, O’Kennedy R. Virus detection: A review of the current and emerging molecular and immunological methods. Front Mol Biosci. 2021;8:637559. 10.3389/fmolb.2021.637559 33959631 PMC8093571

[13] Health, Center for Devices and Radiological. SARS-CoV-2 viral mutations: Impact on COVID-19 tests [homepage on the Internet]. FDA; 2024 [cited 2024 Oct 04]. Available from: https://www.fda.gov/medical-devices/coronavirus-covid-19-and-medical-devices/sars-cov-2-viral-mutations-impact-covid-19-tests

[14] Thermo Fisher Scientific. Clinical conversations. As the SARS-CoV-2 virus evolves, PCR diagnostic and surveillance solutions help stay ahead of viral variants [homepage on the Internet]. 2022 [cited 2025 Apr 14]. Available from: https://www.thermofisher.com/blog/clinical-conversations/as-sars-cov-2-virus-evolves-pcr-diagnostic-and-surveillance-solutions-help-stay-ahead-of-viral-variants/

[15] Pisano MB, Sicilia P, Zeballos M, et al. SARS-CoV-2 genomic surveillance enables the identification of delta/omicron co-infections in Argentina. Front Virol. 2022;2:910839. 10.3389/fviro.2022.910839

[16] Fan G, Jin Y, Wang Q, Yue Y. Assessing the comparability of cycle threshold values derived from five external quality assessment rounds for omicron nucleic acid testing. Virol J. 2023;20:119. 10.1186/s12985-023-02032-z 37291570 PMC10249569

[17] Tembo J, Egbe NF, Maluzi K, et al. Evaluation of SARS-CoV-2 diagnostics and risk factors associated with SARS-CoV-2 infection in Zambia. Int J Infect Dis. 2022;120:150–157. 10.1016/j.ijid.2022.04.017 35427785 PMC9004225

[18] Nyagupe C, De Oliveira Martins L, Gumbo H, et al. SARS-CoV-2 mutations on diagnostic gene targets in the second wave in Zimbabwe: A retrospective genomic analysis. S Afr Med J. 2023;113(3):141–147. 10.7196/SAMJ.2023.v113i3.16762 36876349

[19] Chen Y, Han Y, Yang J, Ma Y, Li J, Zhang R. Impact of SARS-CoV-2 variants on the analytical sensitivity of rRT-PCR assays. J Clin Microbiol. 2022;60(4):e0237421. 10.1128/jcm.02374-21 35341301 PMC9020341

[20] Dip SD, Sarkar SL, Setu MAA, et al. Evaluation of RT-PCR assays for detection of SARS-CoV-2 variants of concern. Sci Rep. 2023;13(1):2342. 10.1038/s41598-023-28275-y 36759632 PMC9910272

[21] Malik YA. Covid-19 variants: Impact on transmissibility and virulence. Malays J Pathol. 2022;44(3):387–396. 36591708

[22] Perez-Guzman PN, Knock E, Imai N, et al. Epidemiological drivers of transmissibility and severity of SARS-CoV-2 in England. Nat Commun. 2023;14(1):4279. 10.1038/s41467-023-39661-5 37460537 PMC10352350

[23] Carabelli AM, Peacock TP, Thorne LG, et al. SARS-CoV-2 variant biology: Immune escape, transmission and fitness. Nat Rev Microbiol. 2023;21(3):162–177. 10.1038/s41579-022-00841-7 36653446 PMC9847462

[24] FIND. FIND evaluations of SARS-CoV-2 molecular tests [homepage on the Internet]. [cited 2025 Apr 14]. Available from: https://www.finddx.org/covid-19/find-evaluations-of-sars-cov-2-assays/find-evaluations-of-sars-cov-2-molecular-tests/

[25] Cepheid. Xpert^®^ Xpress SARS-CoV-2 – FDA emergency use authorization [homepage on the Internet]. [cited 2025 Apr 14]. Available from: https://www.cepheid.com/en-CA/about/sars-cov-2-test-development-information.html

[26] Illumina. iSeq 100 Sequencing System specifications | Key performance parameters [homepage on the Internet]. [cited 2025 Apr 14]. Available from: https://www.illumina.com/systems/sequencing-platforms/iseq/specifications.html

[27] GitLab. TRANSVIHMI / GeVarLi GitLab [homepage on the Internet]. [cited 2022 Dec 05]. Available from: https://forge.ird.fr/transvihmi/GeVarLi

[28] Tzou PL, Tao K, Pond SLK, Shafer RW. Coronavirus resistance database (CoV-RDB): SARS-CoV-2 susceptibility to monoclonal antibodies, convalescent plasma, and plasma from vaccinated persons. PLoS One. 2022;17(3):e0261045. 10.1371/journal.pone.0261045 35263335 PMC8906623

[29] McHugh ML. Interrater reliability: The kappa statistic. Biochem Medica. 2012;22(3):276–282. 10.11613/BM.2012.031 PMC3900052 23092060

[30] Dorta-Gorrín A, Navas-Méndez J, Gozalo-Margüello M, Miralles L, García-Hevia L. Detection of SARS-CoV-2 based on nucleic acid amplification tests (NAATs) and its integration into nanomedicine and microfluidic devices as point-of-care testing (POCT). Int J Mol Sci. 2023;24(12):10233. 10.3390/ijms241210233 37373381 PMC10299269

[31] Safiabadi Tali SH, LeBlanc JJ, Sadiq Z, et al. Tools and techniques for severe acute respiratory syndrome coronavirus 2 (SARS-CoV-2)/COVID-19 detection. Clin Microbiol Rev. 2021;34(3):e00228-20. 10.1128/CMR.00228-20 33980687 PMC8142517

[32] El Jaddaoui I, Allali M, Raoui S, et al. A review on current diagnostic techniques for COVID-19. Expert Rev Mol Diagn. 2021;21(2):141–160. 10.1080/14737159.2021.1886927 33593219

[33] Salazar-Ardiles C, Asserella-Rebollo L, Cornejo C, et al. Molecular diagnostic approaches for SARS-CoV-2 detection and pathophysiological consequences. Mol Biol Rep. 2023;50:10367–10382. 10.1007/s11033-023-08844-0 37817022

[34] Oh C, Sashittal P, Zhou A, Wang L, El-Kebir M, Nguyen TH. Design of SARS-CoV-2 variant-specific PCR assays considering regional and temporal characteristics. Appl Environ Microbiol. 2022;88(7):e0228921. 10.1128/aem.02289-21 35285246 PMC9004361

[35] Negrón DA, Kang J, Mitchell S, et al. Impact of SARS-CoV-2 mutations on PCR assay sequence alignment. Front Public Health. 2022;10:889973. 10.3389/fpubh.2022.889973 35570946 PMC9096222

[36] DaAnGene. Orf1ab Gene SARS-CoV RNA detected, Covid-19 PCR Test Kit | DaAnGene [homepage on the Internet]. [cited 2025 Apr 14]. Available from: https://en.daangene.com/products/covid-19-sars-cov-2-test-kit/

[37] Tombuloglu H, Sabit H, Al-Khallaf H, et al. Multiplex real-time RT-PCR method for the diagnosis of SARS-CoV-2 by targeting viral N, RdRP and human RP genes. Sci Rep. 2022;12(1):2853. 10.1038/s41598-022-06977-z 35181721 PMC8857243

[38] Jindal H, Jain S, Suvvari TK, et al. False-negative RT-PCR findings and double mutant variant as factors of an overwhelming second wave of COVID-19 in India: An emerging global health disaster. SN Compr Clin Med. 2021;3(12):2383–2388. 10.1007/s42399-021-01059-z 34568761 PMC8453462

[39] Marembo T, Chimbunde P, Chipendo T, Munemo C, Manangazira P, Bangure D. Comparison of Real-Q 2019-nCoV and DaAn Gene 2019-nCoV polymerase chain reaction assays for the detection of SARS-CoV-2. J Clin Lab Anal. 2021;36(1):e24161. 10.1002/jcla.24161 34882825 PMC8761419

[40] Fainguem NN, Fokam J, Ngoufack Jagni Semengue E, et al. High concordance in SARSCoV-2 detection between automated (Abbott m2000) and manual (DaAn gene) RT-PCR systems: The EDCTP PERFECT-study in Cameroon. J Public Health Afr. 2022;13(1):2163. 10.4081/jphia.2022.2163 35720798 PMC9202465

[41] Platten M, Hoffmann D, Grosser R, et al. SARS-CoV-2, CT-values, and infectivity – Conclusions to be drawn from side observations. Viruses. 2021;13(8):1459. 10.3390/v13081459 34452325 PMC8402774

[42] Fokam J, Essomba RG, Njouom R, et al. Genomic surveillance of SARS-CoV-2 reveals highest severity and mortality of delta over other variants: Evidence from Cameroon. Sci Rep. 2023;13(1):21654. 10.1038/s41598-023-48773-3 38066020 PMC10709425

[43] DaAnGene. FAQs on COVID-19 testing [homepage on the Internet]. [cited 2025 Apr 14]. Available from: https://en.en.daangene.com/faqs-on-covid-19-testing.html

[44] Mo C, Lo K, He Y, et al. Performance comparison of two nucleic acid amplification systems for SARS-CoV-2 detection: A multi-center study. J Clin Lab Anal. 2022;36(11):e24727. 10.1002/jcla.24727 36196490 PMC9701884

[45] Haga SB. Challenges of development and implementation of point of care pharmacogenetic testing. Expert Rev Mol Diagn. 2016;16(9):949–960. 10.1080/14737159.2016.1211934 27402403 PMC6709578

[46] Hailemariam BW, Zealiyas K, Gutema G, et al. Performances of four nucleic acid amplification tests for the identification of SARS-CoV-2 in Ethiopia. Sci Rep. 2022;12(1):20282. 10.1038/s41598-022-24411-2 36434013 PMC9700788

